# Predictive and prognostic role of early apolipoprotein A‐I alteration in recurrent or metastatic nasopharyngeal carcinoma patients treated with anti‐PD‐1 therapy

**DOI:** 10.1002/cam4.6321

**Published:** 2023-07-06

**Authors:** Bi Jing Xiao, Xiao Xian Sima, Gang Chen, Haimiti Gulizeba, Ting Zhou, Yan Huang

**Affiliations:** ^1^ State Key Laboratory of Oncology in South China, Guangdong Key Laboratory of Nasopharyngeal Carcinoma Diagnosis and Therapy Sun Yat‐sen University Cancer Center Guangzhou China

**Keywords:** anti‐PD‐1 therapy, apolipoprotein A‐I, nasopharyngeal carcinoma, prognosis, recurrent or metastatic stage

## Abstract

**Background:**

The primary objective of this study was to evaluate the predictive and prognostic value of serum lipids in recurrent or metastatic nasopharyngeal carcinoma (R/M NPC) patients received anti‐PD‐1 therapy.

**Materials and Methods:**

Patients treated with anti‐PD‐1 therapy (monotherapy or combined with chemotherapy) from two clinical trials (CAPTAIN and CAPTAIN‐1st study) were included. Serum lipids were measured at baseline and after two cycles of treatment. We examined the impact of both baseline and post‐treatment lipid levels on objective response rate (ORR), progression‐free survival (PFS), and duration of response (DOR).

**Results:**

Of 106 patients, 89 patients (84%) were male. The patients' median age was 49 years. An early elevated (after two cycles of treatment) cholesterol (CHO), low‐density lipoprotein cholesterol (LDL‐C), apolipoprotein A‐I (ApoA‐I), and apolipoprotein B (ApoB) were significantly associated with better ORR. Moreover, early elevated CHO, LDL‐C, and ApoA‐I were also positively correlated with DOR and PFS. Further multivariate analysis showed that only early change in ApoA‐I could independently predict PFS (HR, 2.27; 95% CI, 1.11–4.61; *p* = 0.034). The median PFS for patients with early elevated and reduced ApoA‐I was 11.43 and 1.89 months, respectively. However, baseline lipids levels do not play a significant role in the prognosis and prediction of patients with anti‐PD‐1 treatment.

**Conclusion:**

Collectively, an early elevation in ApoA‐I was correlated with better outcomes for anti‐PD‐1 therapy in patients with R/M NPC, suggesting that clinicians should consider the early alteration of ApoA‐I as a useful marker in treating R/M NPC patients with anti‐PD‐1.

## 1 INTRODUCTION

Nasopharyngeal carcinoma (NPC) is one of the most common malignant tumors in China and Southeast Asia, with the highest incidence in head and neck malignant tumors.^1^, ^2^ The 5‐year overall survival rates are over 90% in patients with early‐stage (stage I and II) NPC. Unfortunately, 5%–11% of NPC patients have metastasis at the time of diagnosis, while another 15%–30% of NPC patients develop local recurrence or disseminated diseases during treatment.^3^ The treatment options for the majority of patients with recurrent or metastatic NPC (R/M NPC) are largely dependent on palliative systemic therapies, and the 5‐year overall survival rates for those patients are less than 50%.^4^

Nowadays, a growing number of studies have used immune checkpoint blockades (ICBs) alone or in combination with chemotherapy as a strategy for the treatment of R/M NPC.^5^, ^6^ However, there are still some patients who cannot benefit from anti‐PD‐1/PD‐L1 immunotherapy. Thus, it is critical to identify appropriate biomarkers that could predict a benefit of ICB therapy for R/M NPC patients.

It is well known that cholesterol accumulation is a common feature of cancer tissues and plays an important role in tumorigenesis, tumor progression, and tumor immunology.^7^, ^8^ Previous studies have demonstrated that hyper‐cholesterolemia was associated with better outcomes in cancer patients treated with an immune checkpoint inhibitor (ICI). As a manifestation of a low‐grade inflammation state, hyper‐cholesterolemia could identify tumors that are more likely to be responsive to immunotherapy.^9^, ^10^ Previous studies have also demonstrated that serum lipids may be a promising marker for predicting the efficacy of ICI therapy in solid tumors including non‐small‐cell lung cancer,^11^, ^12^ colon adenocarcinoma,^13^ melanoma, and renal cell carcinoma.^14^ Yet, it is unknown whether baseline and fluctuations of lipid levels in NPC patients undergoing immunotherapy can predict the prognosis of NPC.

The aim of our study was to investigate the potential role of baseline and dynamic change in serum lipids levels in the prognosis of R/M NPC patients treated with immunotherapy.

## 2 MATERIALS AND METHODS

### 2.1 Study design and patients

Patients in our site (Sun Yat‐sen University Cancer Center) from two studies (CAPTAIN study and CAPTAIN‐1st study) were enrolled for analysis. CAPTAIN is an open‐label, multicenter, single‐arm, phase II study conducted in patients with recurrent or metastatic NPC (R/M NPC) at 8 sites in China (ClinicalTrials. gov identifier: NCT03558191). CAPTAIN‐1st is a randomized, double‐blind, placebo‐controlled, phase III trial conducted at 28 sites in China (ClinicalTrials. gov identifier: NCT03707509). Patients were eligible if they were 18–75 years old, had pathologically confirmed recurrent or metastatic nasopharyngeal carcinoma, had not received previous systemic therapy for recurrent or metastatic disease for CAPTAIN‐1st study and disease progression after at least one prior platinum‐based chemotherapy, and had an Eastern Cooperative Oncology Group performance status of 0 or 1. Patients lacking baseline serum lipid data were excluded from the final analysis. Both two studies were conducted in accordance with the Declaration of Helsinki and the Good Clinical Practice guidelines. All patients provided written informed consent. Our study was approved by the ethics committee.

### 2.2 Procedures

In the CAPTAIN‐1st study, patients received four to six cycles of camrelizumab (200 mg on day 1 of each cycle), gemcitabine (1000 mg/m^2^ on days 1 and 8 of each cycle), and cisplatin (80 mg/m^2^ on day 1 of each cycle) during the induction phase. Subsequently, patients received camrelizumab alone for maintenance on day 1 of every 3‐week cycle until the occurrence of disease progression, unacceptable toxicity, or withdrawal of consent. All patients in CAPTAIN study received 200 mg camrelizumab administered intravenously every 2 weeks on 4‐week treatment cycles for 2 years or until disease progression or intolerable adverse events (AEs) occurred, or other discontinuation criteria were met.

Tumor response was assessed with CT and/or MRI by investigators and an independent review committee according to the Response Evaluation Criteria in Solid Tumors version 1.1 (RECIST 1.1) every 2 cycles in CAPTAIN study, every 6 weeks for the first 16 months, and every 12 weeks thereafter in CAPTAIN‐1st study. The confirmatory scan of treatment response was required for the initial complete and partial response no less than 4 weeks.

### 2.3 Outcomes

The primary endpoint of this study was progression‐free survival (PFS) per RECIST 1.1 assessed by an independent review committee. Secondary endpoints were objective response rate per IRC (ORR) and duration of response (DOR). PFS was defined as the period from randomization to disease progression or death from any cause. ORR was defined as the proportion of patients with complete response or partial response. DOR was defined as the time from the first documented objective response to disease progression or death.

### 2.4 Data collection

The data of fasting serum lipid profiles including total cholesterol (CHO), high‐density lipoprotein cholesterol (HDL‐C), low‐density lipoprotein cholesterol (LDL‐C), triglyceride (TG), apolipoprotein A‐I (ApoA‐I), and apolipoprotein B (ApoB) at different time points (baseline, 2 cycles after initiation of ICIs therapy) were collected. Additionally, patients' demographics and clinical parameters including age, gender, Eastern Cooperative Oncology Group performance status (ECOG PS), histology, smoking history, disease stage, and EBV‐DNA level were also documented. The tumor stage at the diagnosis of NPC was defined based on the National Comprehensive Cancer Network guidelines (Head and Neck Cancers Version 1.2022).

### 2.5 Statistical analysis

The X‐tile program (Yale University School of Medicine)^15^ was used to define the optimal cut‐off values for baseline and fluctuation of lipid parameters including CHO, HDL‐C, LDL‐C, TG, ApoA‐I, and ApoB. Based on the lipids cut‐off value, patients were divided into different groups for further analysis. The Pearson correlation, Chi‐square test, and Fisher exact test were used for the comparison of continuous and categorical variables. Comparisons of ORR between groups were evaluated using a Chi‐square test with continuity correction with a two‐sided 5% significance level. Kaplan–Meier curve with the log‐rank test was used to determine the association between serum lipids and DOR or PFS. Association between baseline characteristics and treatment outcomes was performed using univariate and multivariate Cox proportional hazards regression analysis. Receiver operating characteristic (ROC) curves were used to estimate the sensitivity and specificity of biomarkers by calculating the area under the curve (AUC). A two‐sided *p* value of <0.05 was used to define the statistical significance. All statistical analyses were conducted using SPSS 25 (IBM).

## 3 RESULTS

### 3.1 Characteristics of the study cohorts

**FIGURE 1:**
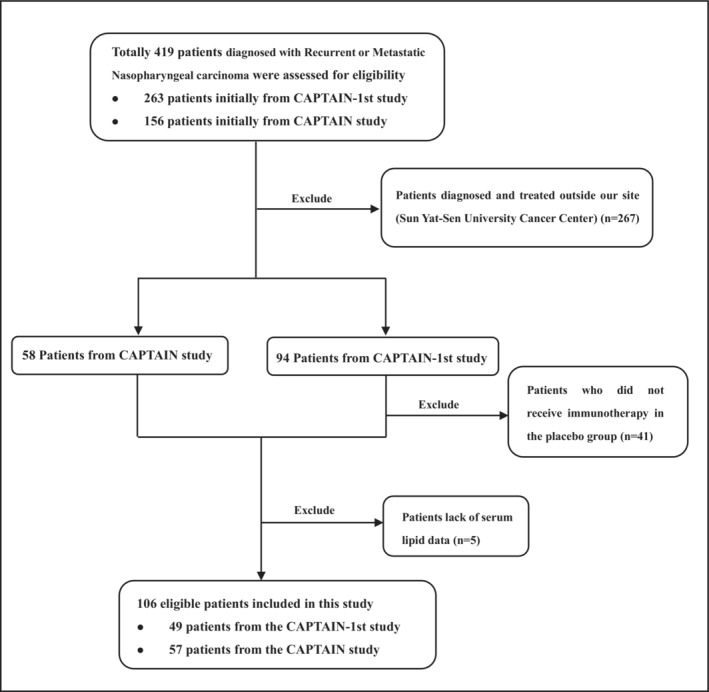
Flow of patients.

A total of 106 R/M NPC patients treated with anti‐PD‐1 monotherapy or a combination of anti‐PD‐1 with chemotherapy (in first‐line or more) were enrolled in the study between July 26, 2018, and March 24, 2020 (Figure 1). Among them, 49 patients were derived from the CAPTAIN‐1st study and 57 patients were derived from the CAPTAIN study, and their data were combined for analysis. Patient ages ranged from 28 to 73 years (median 49 years). All of the patients had a PS of 0–1, and males accounted for 84.0% (*n* = 89). Of these patients, 45.3% were current or former smokers, and 50.9% had liver metastasis. The baseline clinical characteristics of the patients were summarized in Table 1. At the last follow‐up (January 13, 2022), 47 (44.3%) patients died, 7 patients lost to follow‐up and 52 patients remained alive. Median follow‐up time was 23.63 months (range: 0.90–39.33 months).

**TABLE 1:** Patient characteristics at baseline (*n* = 106).

Patient characteristics	No. (%)
*Age, years*	
Median (range)	49 (28–73)
<49	50 (47.2)
≥49	56 (52.8)
*Gender*	
Male	89 (84.0)
Female	17 (16.0)
*ECOG PS*	
0	35 (33.0)
1	71 (67.0)
*Smoking status*	
Never smoker	58 (54.7)
Current or former smoker	48 (45.3)
*Liver metastasis*	
Yes	52 (49.1)
No	54 (50.9)
*EBV‐DNA*	
=0	7 (6.6)
>0	99 (93.4)
*Optimal efficacy*	
PR	58 (54.7)
SD	26 (24.5)
PD	22 (20.8)
*Treatment line*	
1st	49 (46.2)
≥2nd	57 (53.8)
*Treatment type*	
Monotherapy	57 (53.8)
Combination therapy	49 (46.2)

Abbreviations: EBV, Epstein–Barr virus; ECOG PS, Eastern Cooperative Oncology Group performance status; PD, progressive disease; PR, partial response; SD, stable disease.

### 3.2 Baseline serum lipid level and their correlation with clinicopathological characteristics

The mean levels of baseline cholesterol, HDL‐C, LDL‐C, triglyceride, ApoA‐I, and ApoB were 5.15 mmol/L, 1.23 mmol/L, 3.36 mmol/L, 1.41 mmol/L, 1.27 g/L, and 1.09 g/L, respectively (Table 2). The statistical analysis showed that the baseline triglyceride level was significantly correlated with the smoking status (*r* = 0.21, *p* = 0.034). In addition, baseline cholesterol, HDL‐C, and ApoB levels were significantly correlated with ECOG performance status (*r* = 0.29, *p* = 0.003; *r* = 0.21, *p* = 0.029, and *r* = 0.28, *p* = 0.004). Also, baseline cholesterol and ApoA‐I levels were significantly correlated with treatment lines (*r* = 0.27, *p* = 0.006 and *r* = 0.20, *p* = 0.045). There were no correlations of cholesterol, LDL‐C, triglyceride, ApoA‐I, and ApoB with other clinicopathological parameters. Also, baseline HDL‐C levels were not correlated with clinicopathological features (Table S1).

**TABLE 2:** Lipid alterations in all patients.

Lipid	Mean ± SD	*p* Value^b^
*Cholesterol (mmol/L)*		**0.006**
Baseline	5.15 ± 1.16	
After immunotherapy	5.44 ± 1.27	
Difference^a^	0.29 ± 0.11	
*HDL‐C (mmol/L)*		**<0.001**
Baseline	1.23 ± 0.31	
After immunotherapy	1.30 ± 0.31	
Difference^a^	0.07 ± 0.00	
*LDL‐C (mmol/L)*		**0.002**
Baseline	3.36 ± 1.14	
After immunotherapy	3.65 ± 1.19	
Difference^a^	0.29 ± 0.05	
*Triglyceride (mmol/L)*		0.351
Baseline	1.41 ± 0.76	
After immunotherapy	1.47 ± 0.71	
Difference^a^	0.06 ± 0.05	
*ApoA‐I (g/L)*		0.326
Baseline	1.27 ± 0.21	
After immunotherapy	1.29 ± 0.23	
Difference^a^	0.02 ± 0.02	
*ApoB (g/L)*		0.257
Baseline	1.09 ± 0.39	
After immunotherapy	1.12 ± 0.26	
Difference^a^	0.03 ± 0.13	

*Note:* The bold values represent a significant *p* values <0.05.

Abbreviations: ApoA‐I, apolipoproteinA‐I; ApoB, apolipoprotein B; HDL‐C, high‐density lipoprotein cholesterol; LDL‐C, low‐density lipoprotein cholesterol; SD, standard deviation.

^a^ Difference = After immunotherapy lipids‐baseline lipids.

^b^ Compared with paired *t*‐test.

### 3.3 Baseline lipid levels are not associated with prognosis of NPC

Of the 106 patients, there were no patients with complete response, 58 (54.7%) patients with confirmed partial response, and 26 (24.5%) patients with stable disease (Table 1). Median time to response (TTR) was 1.77 months (95% confidence interval [CI] = 1.71–1.83), and the median duration of response (DOR) was 9.27 months (95% CI, 6.15–12.39). Overall, the median PFS was 11.03 months (95% CI, 6.98–15.08). Based on the serum levels of lipids, patients were divided into three groups (low blood lipid group, normal blood lipid group, and high blood lipid group). Statistical analysis showed that there was no significant difference in the correlation between baseline serum lipid levels and ORR (Table S2). Furthermore, PFS was not significantly different among low cholesterol, normal cholesterol, and high cholesterol groups (*p* = 0.814; Table S2). Similarly, PFS was not significantly different among patients with different HDL‐C levels (*p* = 0.055), LDL‐C levels (*p* = 0.974), TG levels (*p* = 0.260), ApoA‐I levels (*p* = 0.198), or ApoB levels (*p* = 0.141; Table S2). These results indicated that baseline lipid levels are not associated with the prognosis of NPC.

### 3.4 Impact of serum lipid alterations after immunotherapy on the efficacy of anti‐PD‐1 treatment

The serum lipid profile in patients before treatment was not significantly different from those after 2 courses (8 [±2] weeks after initiation of anti‐PD‐1 therapy) of treatment (Table 2). The mean levels of cholesterol, HDL‐C, LDL‐C, TG, ApoA‐I, and ApoB in patients with 2 courses of immunotherapy were 5.44 mmol/L, 1.30 mmol/L, 3.65 mmol/L, 1.47 mmol/L, 1.29 g/L, and 1.12 g/L, respectively. The mean ± standard deviation (SD) of alteration in the levels of CHO, HDL‐C, LDL‐C, TG, ApoA‐I, and ApoB after two courses of treatment (comparing to baseline levels) was 0.29 ± 1.04 mmol/L, 0.07 ± 0.27 mmol/L, 0.29 ± 0.93 mmol/L, 0.06 ± 0.62 mmol/L, 0.02 ± 0.24 g/L, and 0.03 ± 0.28 g/L, respectively. X‐tile program was used to obtain the optimal cut‐off values of the alteration in the levels of CHO, HDL‐C, LDL‐C, TG, ApoA‐I, and ApoB. After maximizing the significance by the log‐rank test, these values were set as −0.19 mmol/L, 0.05 mmol/L, −0.63 mmol/L, 0.67 mmol/L, −0.21, and 0.12 g/L, respectively.

**FIGURE 2:**
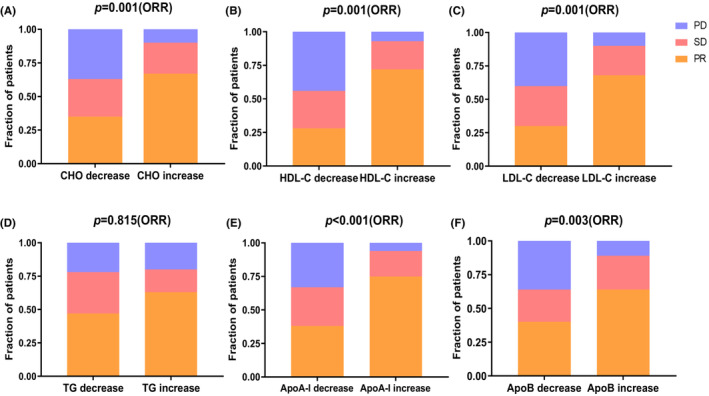
The best responses of each group categorized by the serum lipid fluctuations. PD, progressive disease; PR, partial response; SD, stable disease. *p* values in (A–F) are calculated using the Chi‐squared test for the objective response rate (ORR).

We then analyzed the correlation between the alteration of serum lipid levels and ORR. The results showed that the alterations of cholesterol (*r* = 0.791, *p* = 0.006), HDL‐C (*r* = 0.302, *p* = 0.001), LDL‐C (*r* = 0.704, *p* = 0.004), and ApoA‐I (*r* = 0.133, *p* < 0.001) level were significantly correlated with ORR. ORR in patients with increased ApoA‐I was 67%, which was significantly higher than that (35%) in patients with decreased ApoA‐I (35%) (*p* < 0.001). Similarly, a better ORR for patients with increased CHO (*p* = 0.001), HDL‐C (*p* = 0.001), LDL‐C (*p* = 0.001), and ApoB (*p* = 0.003) was observed compared to those with decreased levels of CHO, HDL‐C, LDL‐C, and ApoB. However, the alteration of TG level was not correlated with ORR (Figure 2).

**FIGURE 3:**
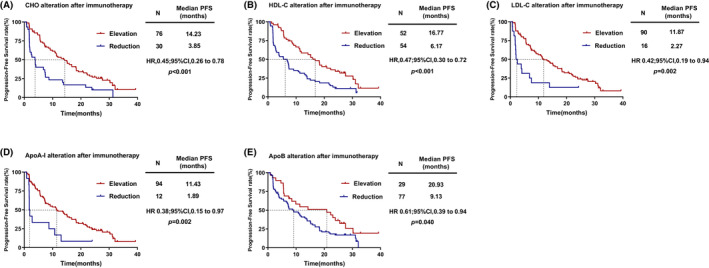
Kaplan–Meier curves for PFS. (A) PFS according to patients with the reduction or elevation of CHO; (B) PFS according to patients with the reduction or elevation of HDL‐C; (C) PFS according to patients with the reduction or elevation of LDL‐C; (D) PFS according to patients with the reduction or elevation of ApoA‐I; (E) PFS according to patients with the reduction or elevation of ApoB.

To further validate this predictive value, we analyzed the correlation between the alteration of serum lipid and PFS. The results showed that patients with increased CHO after two cycles of treatment had significantly longer PFS [HR = 0.45, (95% CI, 0.26–0.78), *p* < 0.001; Figure 3A] than patients with decreased CHO after two cycles of treatment. Similar results were observed for HDL‐C [HR = 0.47, (95% CI, 0.30–0.72), *p* < 0.001; Figure 3B], LDL‐C [HR = 0.42, (95% CI, 0.19–0.94), *p* = 0.002; Figure 3C], ApoA‐I [HR = 0.38, (95% CI, 0.15–0.97), *p* = 0.002; Figure 3D] and ApoB [HR = 0.61, (95% CI, 0.39–0.94), *p* = 0.040; Figure 3E]. However, the alteration of TG was not significantly associated with PFS (*p* = 0.085).

### 3.5 Impact of serum lipid alterations after immunotherapy on DOR

**FIGURE 4:**
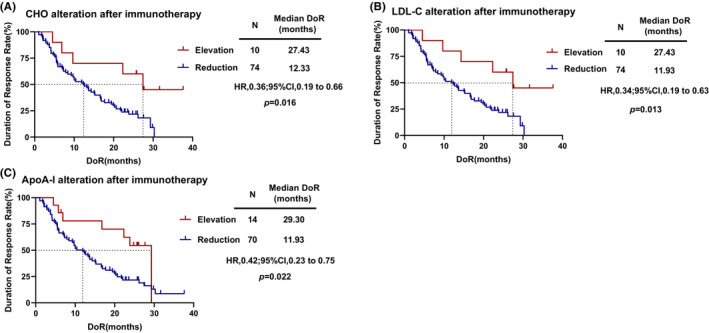
Kaplan–Meier curves for DoR. (A) DoR according to patients with the reduction or elevation of CHO; (B) DoR according to patients with the reduction or elevation of LDL‐C; (C) DoR according to patients with the reduction or elevation of ApoA‐I.

According to Duration of Response (DOR), X‐tile was used to determine the optimal cut‐off values for the serum lipids alteration of CHO, HDL‐C, LDL‐C, TG, ApoA‐I, and ApoB after immunotherapy. Consequently, these values were set as 1.69 mmol/L, 0.38 mmol/L, 1.53 mmol/L, 0.67 mmol/L, 0.26, and 0.28 g/L, respectively. The results showed that the alteration of cholesterol (*r* = 0.233, *p* = 0.032), LDL‐C (*r* = 0.284, *p* = 0.008), and ApoA‐I (*r* = 0.230, *p* = 0.034) level was significantly correlated with DOR. Furthermore, patients with increased cholesterol (HR = 0.36, 95% CI, 0.19–0.66 *p* = 0.016; Figure 4A), LDL‐C (HR = 0.34, 95% CI, 0.19–0.63, *p* = 0.013; Figure 4B) and ApoA‐I (HR = 0.42, 95% CI, 0.23–0.75, *p* = 0.022; Figure 4C) after two cycles of treatment had significantly longer DOR than with decreased serum lipid levels. However, the alteration of HDL‐C (*p* = 0.068), TG (*p* = 0.183), and ApoB (*p* = 0.139) was not significantly associated with DOR.

### 3.6 Univariate and multivariate Cox regression analyses of PFS

To exclude the influence of other variables on PFS, we performed univariate survival analysis based on characteristic factors and the results showed that patients with liver metastasis had shorter PFS than those without liver metastasis (*p* = 0.026). We then included those significant clinicopathological parameters in univariate analysis into the multivariate model. Multivariate analysis showed that liver metastasis (Yes vs. No, HR, 1.62; 95% CI, 1.03–2.55; *p* = 0.037) and a ApoA‐I reduction (reduction vs. elevation, HR, 2.27; 95% CI, 1.11–4.61; *p* = 0.034) after 2 courses of immunotherapy independently predicted shorter PFS (Table 3).

**TABLE 3:** Predictive factors for PFS by univariate and multivariate analysis.

	Univariate analyses		Multivariate analyses	
	HR (95%CI)	*p* Value	HR (95%CI)	*p* Value
*Gender*				
Male vs. female	1.16 (0.66–2.04)	0.598		
*Age (years)*				
<49 vs. ≥49	1.18 (0.77–1.80)	0.445		
*ECOG PS*				
1–2 vs. 0	1.18 (0.76–1.85)	0.461		
*Smoking status*				
Never smoker vs. Current or former smoker	0.91 (0.59–1.39)	0.663		
*EBV‐DNA*				
0 vs. >0	1.19 (0.48–2.96)	0.701		
*Liver metastasis*				
Yes vs. No	1.63 (1.06–2.51)	**0.026**	1.62 (1.03–2.55)	**0.037**
*Cholesterol alteration*				
Reduction vs. elevation	2.28 (1.44–3.59)	<**0.001**	1.23 (0.61–2.50)	0.564
*HDL‐C alteration*				
Reduction vs. elevation	2.18 (1.42–3.35)	<**0.001**	1.51 (0.85–2.66)	0.159
*LDL‐C alteration*				
Reduction vs. elevation	2.43 (1.36–4,34)	**0.002**	1.68 (0.77–3.66)	0.192
*Triglyceride alteration*				
Reduction vs. elevation	1.90 (0.92–3.94)	0.085		
*ApoA‐I alteration*				
Reduction vs. elevation	2.71 (1.43–5.16)	**0.002**	2.27 (1.11–4.61)	**0.034**
*ApoB alteration*				
Reduction vs. elevation	1.67 (1.02–2.73)	**0.040**	1.16 (0.65–2.05)	0.619

*Note*: Values in boldface indicate *p* values <0.05.

Abbreviations: ApoA‐I, apolipoprotein A‐I; ApoB, apolipoprotein B; CI, confidence interval; EBV, Epstein–Barr virus; ECOG PS, Eastern Cooperative Oncology Group performance status; HDL‐C, high‐density lipoprotein cholesterol; HR, hazard ratio; LDL‐C, low‐density lipoprotein cholesterol; PFS, progression‐free survival.

Finally, we plotted receiver operating characteristic (ROC) curves to consider both the specificity and sensitivity of the lipids level. We found that the AUC for the alteration of ApoA‐I level was 0.647 (*p* = 0.042) based on a 0.26 g/L cut‐off (Figure S1), while the alteration of CHO (*p* = 0.066), HDL‐C (*p* = 0.106), LDL‐C (*p* = 0.227), TG (*p* = 0.448) and ApoB (*p* = 0.422) were not statistically significant in the ROC analysis, indicating the prognostic value of the alteration of ApoA‐I after anti‐PD‐1 therapy.

## 4 DISCUSSION

In this study, we explored the role of serum lipid levels in the prognosis of R/M NPC patients with anti‐PD‐1‐based therapy. We found that baseline levels of serum lipids were not correlated with the survival of R/M NPC patients with anti‐PD‐1‐based therapy. However, our study demonstrated that patients with early elevated CHO, HDL‐C, LDL‐C, ApoA‐I, and ApoB after two cycles of treatment had better clinical outcomes compared with those with reduced levels of these lipids. Further multivariate Cox regression analyses verified that the alteration of ApoA‐I after anti‐PD‐1‐based therapy may be an independent prognostic marker of PFS. To the best of our knowledge, this is the first randomized study that evaluated the early variation of serum lipid level and efficacy of immune therapy in R/M NPC patients. Therefore, these findings indicated that serum lipids, particularly ApoA‐I, should be considered when selecting patients for anti‐PD‐1‐based therapy.

Accumulating evidence has shown that the serum lipids level of cancer patients was correlated with many immune biomarkers, including CD3, CD8, CD163, and iNOS,^16^ which could promote the function of effector T cells and potentiate an antitumor program.^17^, ^18^ Moreover, multiple lipids may affect the immune response in vivo through different mechanisms, for example, alterations in the development, phenotype, and activity of leukocytes and the induction of oxidative stress.^19^, ^20^ Several studies indicated that serum lipids could be a promising marker for predicting the efficacy of immune checkpoint inhibitor therapy in solid tumors including non‐small‐cell lung cancer,^11^ colon adenocarcinoma,^13^ melanoma, and renal cell carcinoma.^14^ However, these studies only analyzed the influence of baseline blood lipid levels on the outcomes of cancer patients. In this study, we showed that the baseline serum lipids levels were not significantly correlated with clinical outcomes of R/M NPC patients with anti‐PD‐1‐based therapy. Since the baseline blood lipid level is closely correlated with previous underlying diseases, dietary habits, and the use of lipid‐lowering drugs, it may not be appropriate to use baseline lipids level to predict the prognosis of NPC patients. Therefore, in this study, we analyzed the role of early dynamic changes of blood lipids following two courses of anti‐PD‐1 therapy on the outcome of the treatment.

It is well known that the accumulation of cholesterol is a general feature of tumor tissues.^21^ Thus, the accumulation of lipids in tumor cells could result in lipid deficiency in immune cells. Furthermore, tumor cells are equipped to attenuate the inflammatory activity of T cells and macrophages by promoting free cholesterol efflux, leading to an increase in the proportion of T regulatory cells.^22^ Therefore, increased serum lipid levels may compensate for the lipid deprivation in immune cells in the tumor microenvironment. Previous studies have demonstrated that increased exogenous lipids can potentially restore the activity of lipid‐deficient immune cells in the tumor microenvironment, thereby enhancing antitumor activity.^23^, ^24^ Otherwise, a recent study indicated that 3‐hydroxy‐3‐methyl‐glutaryl‐CoA reductase (HMGCR) and acetyl‐coenzyme A cholesterol acetyltransferase 1 (ACAT1) upregulated and ATP‐binding cassette (ABC)A1 downregulated in the lung adenocarcinoma tumor region, which would induce cholesterol dysregulated cellular export.^25^ The initiation of ICI therapy may cause tumor cell necrosis, then the serum cholesterol levels would increase. Based on these findings, we hypothesized that early alteration of serum lipid levels could be associated with the efficacy of anti‐PD‐1‐based therapy in R/M NPC patients. This hypothesis was supported by our results that patients with an elevated lipid level after anti‐PD‐1 treatment had a better ORR compared with the patients with a decreased lipid level.

We further explored the prognostic role of lipid alteration in PFS. Our results showed that patients with elevated APOA‐I had significantly longer PFS than patients with decreased serum lipid levels. Similarly, we also found patients with increased CHO, HDL‐C, LDL‐C, and ApoB after 2 cycles of anti‐PD‐1 treatment had significantly longer PFS compared with those with decreased levels of these lipids. Moreover, patients with elevated cholesterol, LDL‐C, and ApoA‐I also had significantly longer DOR. Serum lipids including ApoA‐I could suppress tumor growth and metastasis, primarily via up‐regulation of immune infiltration and T‐cell function in the tumor microenvironment.^17^, ^23^ We demonstrated here that elevation of lipid levels is associated with the increased long‐term survival of NPC patients treated with immunotherapy.

Finally, our multivariate analysis showed that ApoA‐I is the best prognostic biomarker (reduction vs. elevation, HR, 2.27; 95% CI, 1.11–4.61; *p* = 0.034) to predict the outcomes of NPC patients treated with anti‐PD‐1 therapy. Therefore, monitoring the dynamic change in blood lipids level, especially the changes in ApoA‐I, is a powerful and cost‐effective method in clinical practice to identify patients who are more likely to benefit from immunotherapy.

There are several limitations to this study. Firstly, our study only enrolled the patients who were treated and followed up at Sun Yat‐sen University Cancer Center. However, all the patients and clinical data were derived from clinical trials with strict quality control and good follow‐up, and this cohort is representative of the overall NPC patient population treated with anti‐PD‐1 therapy at our institution. Secondly, because of the limited sample size, the sample set of this exploratory study was not divided into a training and validation set. Additional validation and in‐depth analysis are required in future research. Finally, the effect of using statin on the efficacy of ICIs was not analyzed in this study. Because our study only enrolled part of patients treated at our institution, the analysis does not attempt to specify a universally applicable cut point of the serum lipid alteration level for derived benefit. We instead highlight a trend of an increase in the benefit of NPC patients with increasing serum lipid levels after anti‐PDI treatment. As a result of variations in different centers, treatments, and informatic methods, a relevant numerical cut point needs to be specific to the methods and distinct to particular clinical situations.

## 5 CONCLUSION

In conclusion, this study demonstrated that serum lipid dynamics could be used for predicting the efficacy of ICIs. Particularly, we demonstrated that early change in ApoA‐I level was associated with the clinical outcomes in R/M NPC patients treated with anti‐PD‐1 therapy. Therefore, monitoring the dynamic changes in serum lipid level is a powerful and cost‐effective biomarker to identify the NPC patients who are most likely to benefit from immunotherapy.

## AUTHOR CONTRIBUTIONS

**Bi Jing Xiao:** Conceptualization (equal); data curation (equal); formal analysis (equal); investigation (equal); methodology (equal); resources (equal); software (equal); validation (equal); writing – original draft (equal). **Xiao Xian Sima:** Conceptualization (equal); data curation (equal); formal analysis (equal); resources (equal); software (equal); supervision (equal); validation (equal). **Gang Chen:** Formal analysis (equal); resources (equal); supervision (equal); validation (equal). **Haimiti Gulizeba:** Formal analysis (equal); supervision (equal); validation (equal). **Ting Zhou:** Conceptualization (equal); data curation (equal); formal analysis (equal); investigation (equal); project administration (equal); validation (equal); writing – original draft (equal); writing – review and editing (equal). **Yan Huang:** Conceptualization (equal); formal analysis (equal); funding acquisition (equal); validation (equal); writing – review and editing (equal).

## FUNDING INFORMATION

This work was supported by Science and Technology Projects in Guangzhou (grant number 202201010958) and Medical Scientific Research Foundation of Guangdong Province of China (A2022292). Both of the agencies have no roles in the design and conduct of the study; collection, management, analysis, and interpretation of the data; preparation, review, or approval of the manuscript; and decision to submit the manuscript for publication.

## CONFLICT OF INTEREST STATEMENT

The authors declare that they have no conflict of interest.

## Supporting information


Figure S1.

Table S1.

## Data Availability

The authors confirm that the data supporting the findings of this study are available within the article.
