# Supplementary material for: Predictive and prognostic role of early apolipoprotein A‐I alteration in recurrent or metastatic nasopharyngeal carcinoma patients treated with anti‐PD‐1 therapy
Source: Cancer Med. 2023 Jul 6;12(16):16918–28. doi: 10.1002/cam4.6321 (PMC10501269; doi:10.1002/cam4.6321)
Supplement: Supplementary file 1 — Figure S1. [file CAM4-12-16918-s001.pdf]

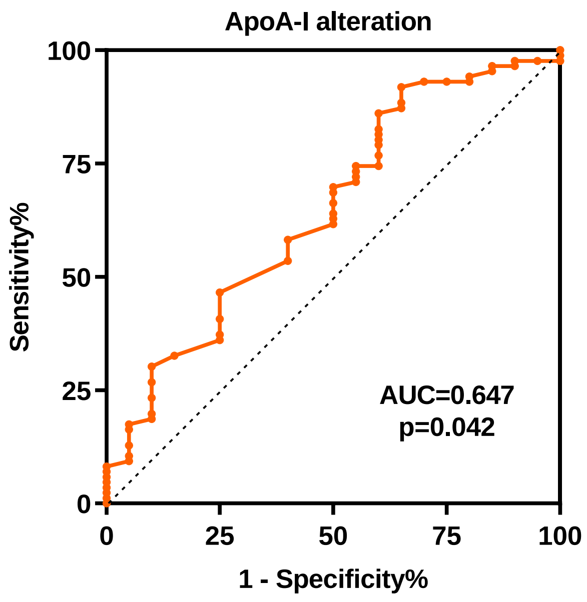

Supplementary Figure 1. Receiver operating characteristic (ROC) curves plotting the predictive value of the alteration of ApoA-I in R/M NPC patients treated with anti-PD-1 therapy.
