# Supplementary material for: Predictive and prognostic role of early apolipoprotein A‐I alteration in recurrent or metastatic nasopharyngeal carcinoma patients treated with anti‐PD‐1 therapy
Source: Cancer Med. 2023 Jul 6;12(16):16918–28. doi: 10.1002/cam4.6321 (PMC10501269; doi:10.1002/cam4.6321)
Supplement: Supplementary file 2 — Table S1. [file CAM4-12-16918-s002.docx]

**Supplementary Table1 Correlation Analysis of Lipids Levels With Clinicopathological Features^Φ^**

|  |  | **CHO** |  | **HDL-C** |  | **LDL-C** |  | **TG** |  | **ApoA-I** |  | **ApoB** |
| --- | --- | --- | --- | --- | --- | --- | --- | --- | --- | --- | --- | --- |
| **Variables** |  | **r *P* value** |  | **r *P* value** |  | **r *P* value** |  | **r *P* value** |  | **r *P* value** |  | **r *P* value** |
| Age | ≤49 vs. >49 | 0.09 0.367 |  | 0.12 0.216 |  | 0.09 0.366 |  | 0.001 0.989 |  | 0.15 0.126 |  | 0.14 0.164 |
| Gender | Male vs. Female | -0.03 0.802 |  | 0.12 0.222 |  | *-*0.09 0.348 |  | -0.06 0.532 |  | 0.09 0.339 |  | -0.07 0.451 |
| Smokers | Yes vs. No | 0.001 0.994 |  | 0.004 0.970 |  | 0.004 0.970 |  | **0.21 0.034** |  | 0.12 0.241 |  | -0.09 0.356 |
| ECOG PS | 0 vs. 1 | **0.29**  **0.003** |  | 0.03 0.780 |  | **0.21** **0.029** |  | 0.06 0.522 |  | 0.01 0.943 |  | **0.28 0.004** |
| EBV DNA | 0 vs. >0 | -0.03 0.759 |  | -0.07 0.472 |  | -0.06 0.554 |  | -0.09 0.349 |  | -0.12 0.211 |  | -0.01 0.906 |
| Treatment line | 1st vs. ≥2nd | **0.27**  **0.006** |  | 0.16 0.113 |  | 0.19 0.052 |  | 0.09 0.353 |  | **0.20 0.045** |  | 0.09 0.346 |
| Liver Metastasis | Yes vs. No | -0.12 0.233 |  | -0.13 0.182 |  | -0.16 0.097 |  | -0.05 0.624 |  | -0.15 0.118 |  | 0.01 0.931 |

Abbreviations: CHO=cholesterol. HDL-C=high-density lipoprotein cholesterol. LDL-C=low-density lipoprotein cholesterol.TG= Triglyceride. ApoA-I=apolipoprotein A-I.

ApoB=apolipoprotein B. ECOG PS=Eastern Cooperative Oncology Group performance status. EBV=Epstein-Barr virus.

**^Φ^**Values in boldface indicate P values <0.05.

**Supplementary Table2 The prognostic value of baseline lipids.**

|  | **N (%)** | **ORR Pvalue** | **Median DOR Pvalue**  **(months)** | **Median PFS Pvalue**  **(months)** |
| --- | --- | --- | --- | --- |
| Baseline CHO  Low Group  Normal Group  High Group | 1(0.9)  74(69.8)  31(29.3) | 0.284  100%  58.1%  45.2% | 0.072  NA  9.67  6.93 | 0.814  NA  11.33  8.73 |
| Baseline HDL-C  Low Group  Normal Group  High Group | 49(46.2)  33(31.1)  24(22.7) | 0.275  55.1%  63.7%  41.7% | 0.256  7.17  12.63  6.9 | 0.055  8.47  18.23  7.43 |
| Baseline LDL-C  Low Group  Normal Group  High Group | 12(11.3)  36(33.9)  58(54.8) | 1.000  58.3%  55.6%  53.3% | 0.398  4.17  7.87  9.73 | 0.974  7.40  11.33  11.03 |
| Baseline TG  Low Group  Normal Group  High Group | 0(0)  80(75.4)  26(24.6) | 0.368  NA  57.5%  46.2% | 0.633  NA  9.67  7.87 | 0.260  11.43  10.77  5.47 |
| Baseline ApoA-I  Low Group  Normal Group  High Group | 43(40.6)  57(53.8)  6(5.6) | 0.857  58.1%  52.7%  5.0% | 0.270  7.17  9.67  6.93 | 0.198  NA  11.43  8.73 |
| Baseline ApoB  Low Group  Normal Group  High Group | 0(0)  62(75.4)  44(24.6) | 0.696  NA  56.5%  52.3% | 0.809  NA  7.87  9.73 | 0.573  NA  10.77  11.03 |

Abbreviations: N=Number. ORR=objective response rate. DOR=duration of Response. PFS=progression-free survival.

CHO=cholesterol. HDL-C=high-density lipoprotein cholesterol. LDL-C=low-density lipoprotein cholesterol.TG= Triglyceride.

ApoA-I=apolipoprotein A-I. ApoB=apolipoprotein B.
